# Editorial: Vitamin C from bench to bedside

**DOI:** 10.3389/fnut.2024.1406342

**Published:** 2024-04-29

**Authors:** Liliana Mititelu-Tartau, Maria Bogdan, Manuela Ciocoiu

**Affiliations:** ^1^Department of Pharmacology, Faculty of Medicine, “Grigore T. Popa” University of Medicine and Pharmacy, Iasi, Romania; ^2^Department of Pharmacology, Faculty of Pharmacy, University of Medicine and Pharmacy, Craiova, Romania; ^3^Department of Pathophysiology, Faculty of Medicine, “Grigore T. Popa” University of Medicine and Pharmacy, Iasi, Romania

**Keywords:** vitamin C, immunity, antioxidant, inflammation, dietary supplements

Vitamin C, also known as ascorbic acid, has long held a place in the pantheon of essential nutrients (1, 2). From its discovery in the early 20th century to its ubiquitous presence in health food stores today, its journey from bench to bedside has been one of intrigue, controversy, and groundbreaking discovery (3, 4). Vitamin C serves as a potent antioxidant, scavenging free radicals and protecting cells from oxidative damage (5, 6). Also, it contributes to the reduction of tiredness and fatigue, to normal functioning of the nervous system, to the regeneration of the reduced form of vitamin E, and increases iron absorption.^1^ Moreover, it plays a crucial role in collagen synthesis, wound healing, and immune function (7–10). These fundamental discoveries have laid the groundwork for understanding the broader implications of vitamin C in human health.

Experimental studies have illuminated the potential therapeutic effects of vitamin C in various diseases (11–13). From its purported anti-cancer properties to its role in mitigating the symptoms of the common cold, researchers have delved deep into the biological pathways through which vitamin C exerts its effects (14). Furthermore, emerging evidence suggests that vitamin C may play a role in combating inflammation, cardiovascular disease, and neurodegenerative disorders (15–18). In recent years, the field of nutritional psychiatry has emerged as a promising avenue for understanding the complex interplay between diet and mental health (Chen et al.) (19).

In infectious diseases, vitamin C has been the subject of intense scrutiny, particularly in the context of the common cold and more recently, the COVID-19 pandemic (20, 21). Various researches suggest that adequate vitamin C intake may lower the risk of developing certain acute and chronic diseases by supporting immune function and reducing inflammation (22). Considering the COVID-19 pandemic, interest in the potential role of vitamin C in bolstering immune health has surged (23, 24).

In this Research Topic there are seven papers published, covering the above-mentioned aspects.

Luo et al. conducted a meta-analysis of randomized controlled trials to investigate the impact of intravenous vitamin C as an adjunctive therapy in patients with sepsis. Their analysis incorporated ten studies comprising 1,426 patients. The findings revealed that the administration of vitamin C to sepsis patients did not lead to a significant reduction in short-term mortality, length of Intensive Care Unit (ICU) stay, or the Sequential Organ Failure Assessment (SOFA) score. However, it was noted that vitamin C could decrease the duration of vasopressor usage, with potential greater benefits observed in sepsis patients from developing countries compared to those from developed nations.

A noteworthy case report by Lu et al. described a rare occurrence involving a 25-year-old male patient without underlying health conditions. The patient presented with progressively severe pain and ecchymoses in both lower extremities over a three-week period during the COVID-19 pandemic and was subsequently diagnosed with scurvy. Given the challenge of diagnosing scurvy due to its nonspecific clinical manifestations, early recognition and prompt treatment are crucial to prevent life-threatening complications in the advanced stages of the disease. This case report emphasizes the importance of being alert for nutritional deficiencies.

Sun et al. devised a protocol for a prospective, randomized, double-blinded, placebo-controlled study aimed at investigating the impact of high-dose intravenous vitamin C treatment on the prognosis of patients with moderately severe and severe acute pancreatitis.

Ding et al. conducted a cross-sectional analysis utilizing the National Health and Nutrition Examination Survey 2017–2018 dataset. Their study involved 5,380 adults from the USA aged ≥20 years and examined the association between vitamin C levels and hs-CRP. Through multiple regression models, stratified analysis, and curve fitting, they assessed the linear and nonlinear relationship between plasma vitamin C and serum hs-CRP. The findings suggested potential cardioprotective and anti-inflammatory effects associated with optimal vitamin C intake, as well as a non-linear negative correlation between vitamin C levels and hs-CRP in adults. Nonetheless, further investigation is warranted to better understand the underlying mechanisms.

Chiscano-Camón et al. conducted a prospective observational study at a single center, involving 43 patients diagnosed with severe COVID-19 pneumonia. They assessed the plasma levels of vitamin C on days 1, 5, and 10 following admission to ICU. The study revealed that vitamin C concentrations were undetectable upon ICU admission in 86% of patients, and this trend persisted throughout the observation period.

Chen et al. explored the relationship between serum vitamin C concentration and the prevalence of depression among adults in the United States. Their research utilized data from the National Health and Nutrition Examination Survey 2017–2018, encompassing 3,404 participants. The results unveiled a negative correlation between serum vitamin C levels and depression, underscoring a noteworthy association between higher levels of serum vitamin C and a reduced prevalence of depressive symptoms.

The case-control pilot study by Noronha et al. analyzed CpG sites related both to the relative abundance of specific bacteria phyla and vitamin C ingestion, in 6 women aged 30.8 ± 2.2. Their analysis revealed that increased vitamin C intake corresponded to a higher relative abundance of Actinobacteria, showing a positive correlation between these factors. Among participants with elevated vitamin C intake, 207 CpG sites were consistently associated with both vitamin C consumption and Actinobacteria abundance. Hypomethylated sites were linked to glucose homeostasis and general cellular metabolism, while immune function was associated with both hypo- and hypermethylated sites. These findings provide deeper insights into the complex interaction among microbiota, nutrients, DNA methylation, and immune response.

From the laboratory bench to the patient's bedside, the journey of vitamin C has been marked by both triumphs and challenges. While its therapeutic potential in various diseases continues to be investigated, the importance of maintaining adequate vitamin C levels for overall health and wellbeing cannot be overstated. As we navigate the complex interplay between diet, supplementation, and disease prevention, it is essential to approach the role of vitamin C with scientific rigor and an open mind. By embracing the potential of vitamin C and leveraging the insights gleaned from bench research, we can pave the way for innovative approaches to healthcare that prioritize prevention, personalized medicine, and holistic wellbeing.

## Author contributions

LM-T: Writing – original draft. MB: Writing – original draft. MC: Writing – review & editing.

## Footnotes

^1^https://ec.europa.eu/food/food-feed-portal/screen/health-claims/eu-register (accessed 31 March, 2024).
